# Recombinant Reg3β protein protects against streptozotocin-induced β-cell damage and diabetes

**DOI:** 10.1038/srep35640

**Published:** 2016-10-21

**Authors:** Chen Luo, Lu-Ting Yu, Meng-Qi Yang, Xiang Li, Zhi-Yuan Zhang, Martin O Alfred, Jun-Li Liu, Min Wang

**Affiliations:** 1 School of Life Science & Technology, China Pharmaceutical University, Nanjing, China; 2 State Key Laboratory of Nature Medicines, China Pharmaceutical University, Nanjing, China; 3 Fraser Laboratories for Diabetes Research, Department of Medicine, McGill University Health Centre, Montreal, Canada

## Abstract

Regenerating genes (Reg) have been found during the search for factors involved in pancreatic islet regeneration. Our recent study discovered that pancreatic β-cell-specific overexpression of Reg3β protects against streptozotocin (Stz) -induced diabetes in mice. To investigate its potential roles in the treatment of diabetes, we produced a recombinant Reg3β protein and provided evidence that it is active in promoting islet β-cell survival against Stz- triggered cell death. Though ineffective in alleviating preexisting diabetes, pretreatment of recombinant Reg3β was capable of minimizing the Stz-induced hyperglycemia and weight loss, by preserving serum and pancreatic insulin levels, and islet β-cell mass. No obvious changes were observed in the rate of cell proliferation and hypertrophy in α- or acinar-cells after treatment with recombinant Reg3β. The underlying mechanism of Reg3β-mediated protection seems to involve Akt activation which upregulates Bcl-2 and Bcl-xL levels and consequently promotes cell survival.

Regenerating genes (Reg) were first discovered during the search for factors involved in pancreatic islet regeneration in 90% depancreatized rats^1^. They constitute a family of secreted polypeptides with similarities not only in open reading frames, but also extending to promoter sequences, suggesting their analogous bioactivities^2^. The calcium dependent lectin (C-type lectin) domain at the carboxyl terminus of the Reg proteins is considered to be essential for carbohydrate recognition which in turn activates multiple downstream signals. Attention has been paid to the therapeutic potential of Reg proteins due to their enhancement of cell proliferation, neogenesis and survival^3^.

Insufficient islet β-cell mass and impaired islet function are the primary causes of type 1 diabetes (T1D) and critical elements involved in type 2 diabetes (T2D). Various growth factors have been found so far to promote islet β-cell growth and/or survival^4^, yet few have been proven potent enough for the treatment of diabetes. A bioactive pentadecapeptide (104–118), derived from islet neogenesis-associated protein (INGAP, of golden hamster) and highly homologous to mouse Reg3δ, has been found to be efficacious in clinical trials for diabetic treatment^5^. Other Reg proteins have been found to be effective in stimulating β-cell proliferation and regeneration in various animal models^2^,^3^. Taken together, this evidence strongly suggests the potential usefulness of Reg proteins in defending against or even alleviating the development of diabetes.

Recently, the diabetic-resistant effect of pancreatic specific IGF-I deficiency (PID) raised our research interests. IGF-I is a well-known growth factor that stimulates pancreatic islet development and growth. However, the PID mice exhibited a strong resistance to Stz-induced diabetes^6^. Using a whole genome microarray, we found that the lack of IGF-I activated the expression of other genes, chief among them were the Regs. Many studies have evidenced that Reg1 promotes pancreatic islet β-cell proliferation, regeneration and survival, either by the manner of endogenous overexpression or exogenous protein administration^7^,^8^,^9^. In addition to Reg1, the expression of Reg2 and Reg3β genes was significantly upregulated in the pancreas of PID mice^10^. To reveal their possible contribution to the protective effect, we thereafter developed two mouse models with pancreatic-specific overexpressed Reg2 and Reg3β. Interestingly, acinar overexpression of Reg2 offered no protection while islet-specific Reg3β predominantly ameliorated the hyperglycemia and body weight reduction caused by Stz^11^,^12^. Given this result, Reg3β was chosen for the preparation of recombinant protein and its effectiveness in treating diabetes was assessed in the present study. The expression of Reg3β gene is normally detectable not only in pancreatic acinar-cells but also in islet α-cells^13^, and was strengthened in the islets from patients with new-onset T1D^14^. However, how the increase of Reg3β protein expression affects insulin-producing β-cells is still unclear. Whether recombinant Reg3β protein can be employed as a therapeutic agent in the treatment of diabetes, has yet to be verified.

We have recently built an engineered *E*.*coli* system to produce bioactive recombinant Reg3β protein^15^. In the present study, we present original evidence that recombinant Reg3β protein enhanced islet β-cell survival *in vitro* and defended against Stz-induced diabetes in mice. On the other end, our results failed to suggest any alleviating effect on preexisting diabetes. The underlying mechanism of this protection could be contributed to Akt activation and increased levels of Bcl-2 and Bcl-xL which consequently lead to a resistance to cell death.

## Results

### Production of recombinant Reg3β protein

**Figure 1:**
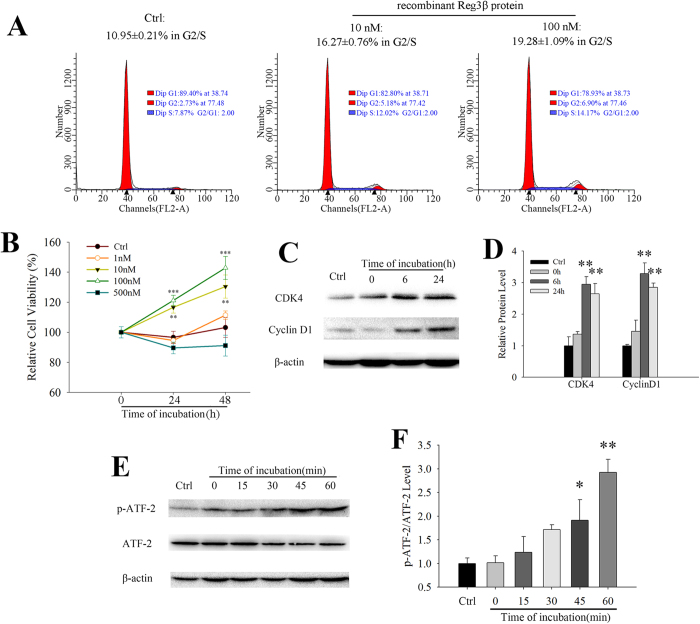
Recombinant Reg3β protein is active in promoting MIN6 cell proliferation. (**A**) Cell cycle analysis of the MIN6 cells treated or untreated with recombinant Reg3β protein. Cells were incubated in the medium containing 1% FBS with different concentrations of this polypeptide and harvested at 24 h for flow cytometry. G2/S indicated the summation of the cells in S phase and G2 phase. N = 3. B. MTT test of the MIN6 cells treated with increasing concentrations of recombinant Reg3β protein. N = 6; **p < 0.01, ***p < 0.001 vs. Ctrl using One Way ANOVA. (**C**,**D**) Western blots and densitometry quantification of the CDK4 and cyclin D1 levels. Cells were incubated with 100 nM recombinant Reg3β protein in the medium containing 1% FBS and harvested at each indicated time points. N = 3; **p < 0.01 vs. Ctrl using One Way ANOVA. (**E**,**F**) Western blots and densitometry analysis of the ATF-2 phosphorylation. Cells were treated with 100 nM Reg3β and then harvested at each of the indicated time points. Densitometry of the ATF-2 phosphorylation was corrected with its total protein. Some blots were edited for better (clear cut) representation and the full-length blots are included in the Supplementary Information file. N = 3; *p < 0.05 and **p < 0.01 vs. Ctrl using One Way ANOVA.

The recombinant Reg3β protein was yielded with a purity of ≥95%, as identified by SDS-PAGE and HPLC methods^15^. To verify its natural bioactivity, we used the MTT assay with increasing concentrations of recombinant protein. As expected, recombinant Reg3β was capable of stimulating MIN6 cell proliferation in a dose-dependent manner, in which 10 ~ 100 nM of recombinant protein was suitable to accelerate cell replication, while a relative higher dose of 500 nM inhibited cell replication [Fig. 1B]. This is in line with the common feature of many growth factors, and indicates that the recombinant Reg3β protein used in the following experiments was biologically active.

### Recombinant Reg3β protein promotes cell cycle progression

The influence of recombinant Reg3β protein on the cell cycle was further examined using flow cytometry in MIN6 cells. After a 24 h starvation period, the portion of the cells in G2/S phase was found to be 10.95 ± 0.21%, and increased to 16.27 ± 0.76% and 19.28 ± 1.09% when treated with 10 and 100 nM recombinant Reg3β, respectively [Fig. 1A]. The levels of CDK4 and cyclin D1 that drive cells over the G1/S checkpoint were also detected to be upregulated by approximately 3- and 3.5-fold, respectively 6 h after the treatment [Fig. 1C,D]. The level of phosphorylated ATF-2 was found to be tripled at 60 min. post treatment [Fig. 1E,F], suggesting that the ATF-2 activation could be an upstream signal of cyclin D1 expression. As recombinant Reg3β protein triggered Akt phosphorylation^15^, the proliferation promoting effect could be mediated by the Akt/ATF-2 pathway and enhanced cyclin D1 expression, consequently driving cells entering G2/S phase with DNA synthesis and preparation for mitosis^7^.

### Recombinant Reg3β protein ameliorates Stz-induced cell death

**Figure 2:**
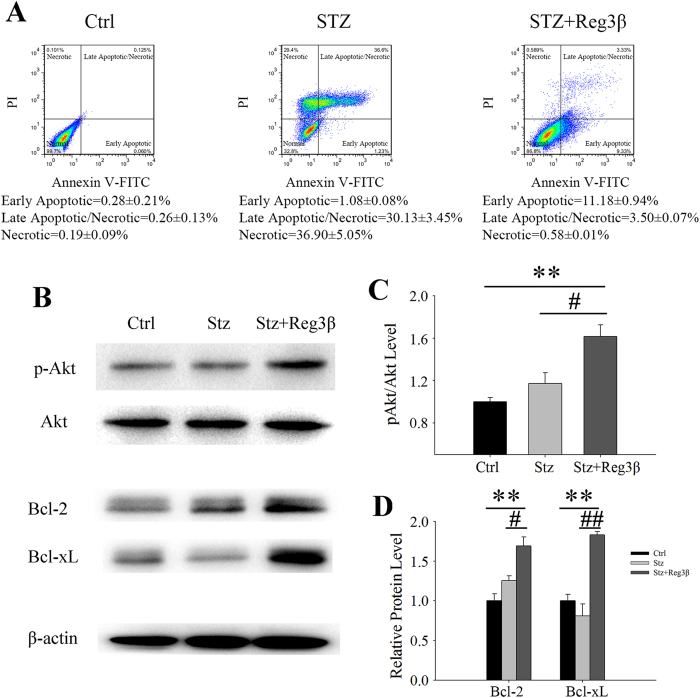
Recombinant Reg3β protects MIN6 cells against Stz-induced cell death. (**A**) Diagram of the flow cytometry of MIN6 cells labeled with annexin-V-FITC and PI. Cells were pretreated with 100 nM recombinant Reg3β protein and then 10 mM Stz was added. Following a 24 h of incubation period, the cells were then harvested for flow cytometry. Cells treated with PBS were set as the Control. N = 3. (**B**) Western blots of the phosphorylated Akt and Bcl-2 and Bcl-xL levels. (**C**) Densitometry quantification of the phosphorylated level of Akt corrected by total Akt, and the protein levels of Bcl-2 and Bcl-xL corrected by β-actin. Some blots were edited for better representation and the full-length blots are included in the Supplementary Information file. N = 3; **p < 0.01 vs. Ctrl, ^#^*p* < 0.05 and ^##^*p* < 0.01 vs. Stz group using One Way ANOVA.

The effect of recombinant Reg3β protein on Stz-induced MIN6 cell death was determined using flow cytometry, Annexin V-FITC and PI double staining. As shown in Fig. 2A, 30.13 ± 3.45% of the total cells found in the double-positive phase in the Stz group was considered to be of late apoptotic or necrotic cells. Meanwhile, cells in the PI single-positive phase, accounting for 36.9 ± 5.05%, were counted as necrotic^16^. Together, around 70% of the cells were undergoing severe cellular stress that could be rescued by the co-incubation with recombinant Reg3β. Despite an increase in the early apoptotic cells to 11.18 ± 0.94%, the sum of the late apoptotic and necrotic cells in the Stz + Reg3β group was drastically reduced to approximately 4%. As previously reported by our group and others, in pancreatitic mice Reg proteins upregulate Bcl-2 and Bcl-xL expression to resist cell death^17^,^18^, and their regulated signaling of Akt kinase. As shown in Fig. 2B–D, a 1.8- and 1.9-fold increase of the Bcl-2 and Bcl-xL levels and a 1.6-fold elevation of the phosphorylated Akt were detected in the Stz + Reg3β group. Though slight increases were also observed in the Stz group, there was no statistical significance. Thus, the protective effect of recombinant Reg3β could be mediated by Akt phosphorylation and upregulated Bcl-2 and Bcl-xL expression, consequently maintaining mitochondrial integrity to resist cell death.

### Recombinant Reg3β protein pretreatment alleviates Stz-induced diabetes in mice

**Figure 3:**
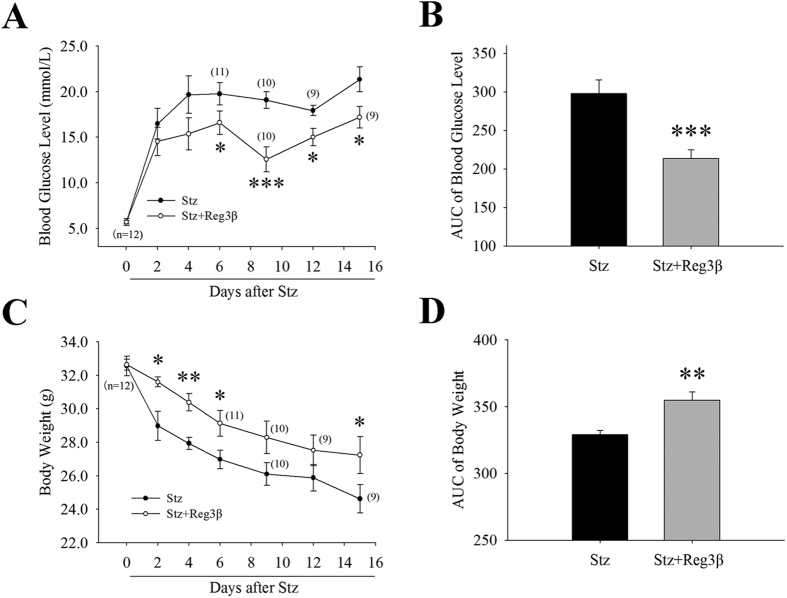
Recombinant Reg3β protein pretreatment alleviates Stz-induced diabetes in mice. (**A**) Detection of the blood glucose level in the Stz and Stz + Reg3β groups within 15 d after being diabetic. N = 9–12; *p < 0.05 and ***p < 0.001 vs. Stz group using t-test. (**B**) AUC statistics of the blood glucose curves in panel A. N = 9; ***p < 0.001 vs. Stz group using t-test. (**C**) Measurement of the weight loss in the Stz and Stz + Reg3β groups within 15 d after being diabetic. N = 9–12; *p < 0.05 and **p < 0.01 vs. Stz group using t-test. (**D**) AUC statistics of the body weight curves in panel C. N = 9; **p < 0.01 vs. Stz group using t-test.

To explore the protective and therapeutic roles of recombinant Reg3β protein in Stz-induced diabetic mice, the protein was introduced via the tail vein anterior or posterior to the Stz injection site. As shown in Fig. 3A, a single injection of Stz at a relative high dose of 150 mg/kg sharply heightened blood glucose to a severe hyperglycemic level of ≥20 mmol/L. Mice pretreated with recombinant Reg3β suffered only mild hyperglycemia at ≤20 mmol/L. Although normoglycemia was not achieved, a statistical difference was found between the Stz and Stz + Reg3β groups [Fig. 3A]. At the same time, acute weight loss of 8 g within 15 d was found in the Stz group, which was effectively alleviated by the pretreatment of recombinant Reg3β [Fig. 3C]. Groups treated with only PBS or recombinant Reg3β exhibited normal blood glucose and body weight [Supplymentary Fig. 1]. When the area under the curve (AUC) using a trapezoidal method was measured for blood glucose and body weight, prominent significances were found between the Stz and Stz + Reg3β groups [Fig. 3B,D].

**Figure 4:**
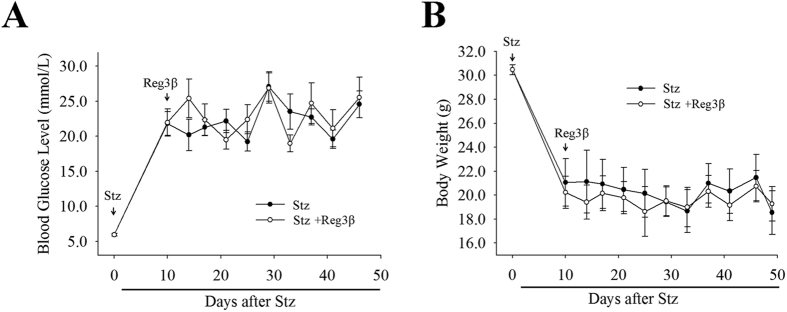
Recombinant Reg3β protein failed to alleviate preexisting diabetes induced by Stz. (**A**) Detection of the blood glucose level in the Stz and Stz + Reg3β groups within 50 d after 5-injections of low-dose Stz. Recombinant Reg3β was given to mice on the 9^th^ day after the first injection of Stz. (**B**) Measurement of the weight loss in mice in the Stz and Stz + Reg3β groups. For A and B, N = 12; no significant difference was found between these groups using t-test.

We performed another set of experiments to determine whether recombinant Reg3β protein can alleviate preexisting diabetes. To develop a stable hyperglycemic model, Stz, at a dose of 50 mg/kg per day, was given for 5 consecutive days, which led to a severe hyperglycemia, ~20 mmol/L, from the 9^th^ day following the first injection till the end of this experiment. However, no statistical significance in glycemia or body weight was found between the Stz and Stz + Reg3β groups [Fig. 4A,B]. Neither of their AUCs showed any significant difference as well [Supplymentary Fig. 2]. Thus, we came to the conclusion that the administration of exogenous recombinant Reg3β protein could protect against Stz-induced diabetes, yet whether it plays a role in curing the preexisting diabetes requires more discussion.

### Recombinant Reg3β protein pretreatment preserves insulin content and islet β-cell mass

**Figure 5:**
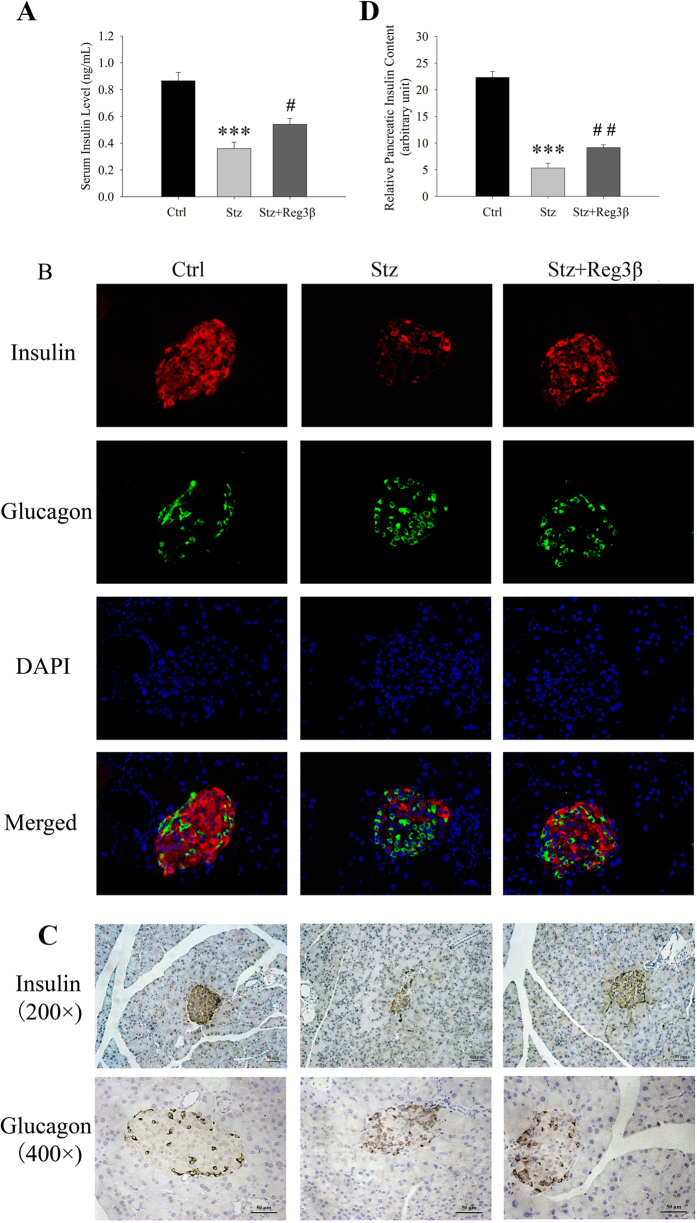
Recombinant Reg3β protein pretreatment preserves the serum insulin level and islet β-cells. (**A**) Changes in serum insulin levels in the Control, Stz and Stz + Reg3β groups 15 d after the Stz injection. N = 5–9; ***p < 0.001 vs. Ctrl, ^#^*p* < 0.05 vs. Stz group using One Way ANOVA. (**B**) Immunofluorescence stained with insulin and glucagon in the Control, Stz and Stz + Reg3β groups. Cell nuclei were labeled with DAPI. A representative image is illustrated from each group. (**C**) Immunohistochemistry stained with insulin or glucagon. A representative image is illustrated and the results of other islets in each group were similar. The quantification and statistical analysis was presented in Table 1. (**D**) Changes in the relative pancreatic insulin contents among these three groups by densitometry quantification. N = 5–9; ***p < 0.001 vs. Ctrl, ^##^*p* < 0.01 vs. Stz group using One Way ANOVA.

To explore how the pretreatment of recombinant Reg3β protein helped resist hyperglycemia and body weight loss, whole blood serum and pancreatic sections were obtained for serum and pancreatic insulin determination and histopathological examination. As shown in Fig. 5A, an insufficient serum insulin level was detected in the Stz group (~0.3 ng/mL) vs. in the Control group (~0.8 ng/mL). Following pretreatment with recombinant Reg3β, serum insulin levels were significantly rescued to ~0.5 ng/mL. Immunofluorescent-staining showed that the insulin-producing β-cells in the Control group occupied a majority of the islet area, whereas staining was drastically diminished by treatment with Stz. The lack of insulin could be due to the loss of β-cell integrity which eventually leads to cell death. In the Stz + Reg3β group, this decrease was largely alleviated, and thus it is likely that the recombinant Reg3β protein preserved islet β-cell mass [Fig. 5B]. Immunohistochemical staining showed that most of the islets from the Stz group underwent insulitis where a considerable number of the islet cells lacked insulin. This observation was also prominently attenuated by the administration of recombinant Reg3β [Fig. 5C]. Densitometry quantification of the relative pancreatic insulin contents in the Control, Stz and Stz + Reg3β groups were estimated as 22.3 ± 1.1, 5.3 ± 0.9 and 9.2 ± 0.5, respectively, in an arbitrary unit of 1000 × IOD/mm^2^ pancreatic area [Fig. 5D].

The measurements of β- and α-cell mass corrected for body weight are presented in Table 1. The average islet size (total insulin-stained area divided by the number of insulin-positive clusters in the whole pancreas) and the islet density (number of the insulin-positive clusters per squared centimeter in the whole pancreatic area) were also calculated. Intriguingly, the islet density slightly increased with the Stz treatment, but was unlikely affected by the treatment of recombinant Reg3β [Table 1]. These additional “islets” could come from the newly developed or transdifferentiated small insulin-producing cell clusters caused by β-cell damage^19^. No statistical significance was observed between the Stz and Stz + Reg3β groups, though the protective tendency was obvious. These data may be due to the presence of some mega islets and odd sectioning.

### Recombinant Reg3β protein has no effect on islet α- or exocrine acinar-cells

As shown in Fig. 5B,C, islets appeared to suffer from Stz-induced damage, and the number of insulin-producing β-cells was drastically decreased resulting in α-cells taking over more areas within the same islets. Furthermore, the α-cells, which normally distribute in the periphery of the islets, migrated towards the central part of the cell [Fig. 5B,C], which is commonly observed after β-cell damage^20^. The statistical analyses of α-cell percentage/islet and α-cell mass/pancreas are presented in Table 1. Despite an increase in α-cell percentage/islet, statistical analysis showed no significant change in α-cell mass/pancreas by the administration of recombinant Reg3β.

Immunofluorescent Ki67 staining was also performed to reveal whether this treatment led to undesired α- and/or acinar-cell growth. The Ki67 signal however, was barely detected in whole pancreas, suggesting no effect of recombinant Reg3β on α- or acinar-cell proliferation [Supplymentary Fig. 3]. Very few Ki67 signals were captured, while most of them were inactive, owing to their cytoplasmic distribution. No change in average acinar-cell size was found between groups, suggesting that treatment with recombinant Reg3β protein has no effect on acinar-cell growth [Supplymentary Fig. 4].

### Recombinant Reg3β protein resists Stz-induced β-cell death with increased Bcl-2 level

**Figure 6:**
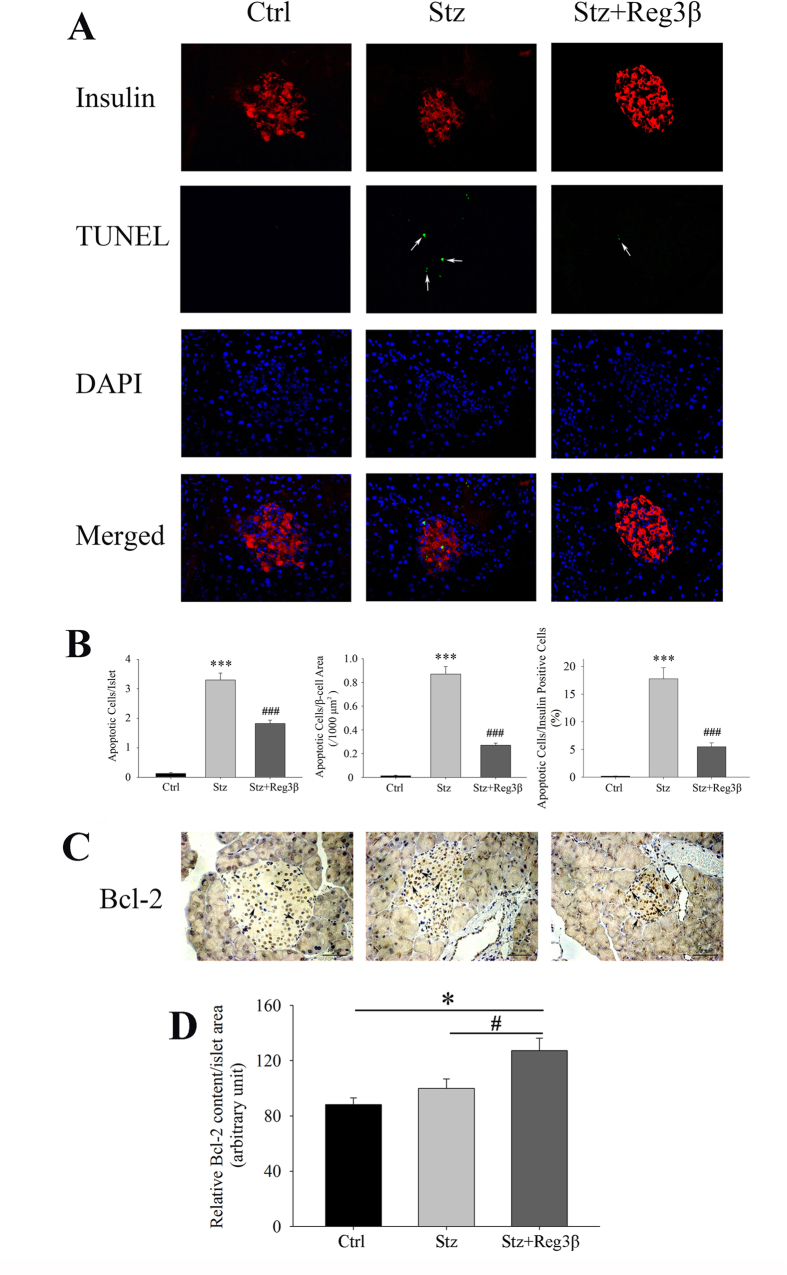
Recombinant Reg3β protein pretreatment enhances β-cell survival with increased Bcl-2 content. (**A**) Immunofluorescence stained with insulin and TUNEL of the pancreatic sections. Arrows: TUNEL apoptotic signals. A representative image was illustrated for each group. (**B**) Quantification of the apoptotic cells in the three groups in Panel A. N = 5; ***p < 0.001 vs. Ctrl, ^###^*p* < 0.001 vs. Stz group using One Way ANOVA. (**C**) Bcl-2 immunohistochemistry of the pancreatic sections. Arrows: Bcl-2. A representative image is illustrated from each group. (**D**) Densitometry quantification of the Bcl-2 content in islets of Panel C. N = 5–9; *p < 0.05 vs. Ctrl, ^#^*p* < 0.05 vs. Stz group using One Way ANOVA.

To demonstrate a direct cytoprotective effect, we monitored acute islet cell apoptosis, as induced by Stz, and the effect of recombinant Reg3β pretreatment. As shown in Fig. 6A,B, we detected positive TUNEL staining in insulin-labeled cells 2 d following Stz injection, indicating apoptotic β-cells. The β -cell area in Stz-treated mice was measured to be approximately 3.3/islet or 0.87/1000 μm^2^, whereas in the Stz + Reg3β group, the β-cell area was significantly reduced to 1.8/islet or 0.27/1000 μm^2^, suggesting that pretreatment with Reg3β partially inhibited apoptosis [Fig. 6A,B]. As high doses of Stz can lead to massive cell necrosis in addition to apoptosis^21^, our observation suggests that treatment with Reg3β prevents diabetes in part by protecting β-cell death. At 15 d following Stz treatment, TUNEL staining became too weak to detect any meaningful change in the different groups (data not shown).

As a constitutively expressed membrane protein associated with the perinuclear membrane, ER and mitochondria, Bcl-2 protein was found detectable in all three groups [Fig. 6C]. With Stz treatment, the expression of Bcl-2 appeared to be upregulated, but was relatively lower than its expression in the Stz + Reg3β group. The densitometry quantification of the relative islet Bcl-2 content revealed a significant 1.4-fold increase in the Stz + Reg3β group, as compared with the Control group. Despite a 1.1-fold rise in the Stz group, there was no significant difference [Fig. 6D].

### Recombinant Reg3β protein protects primary islet cells from Stz-induced cell death

**Figure 7:**
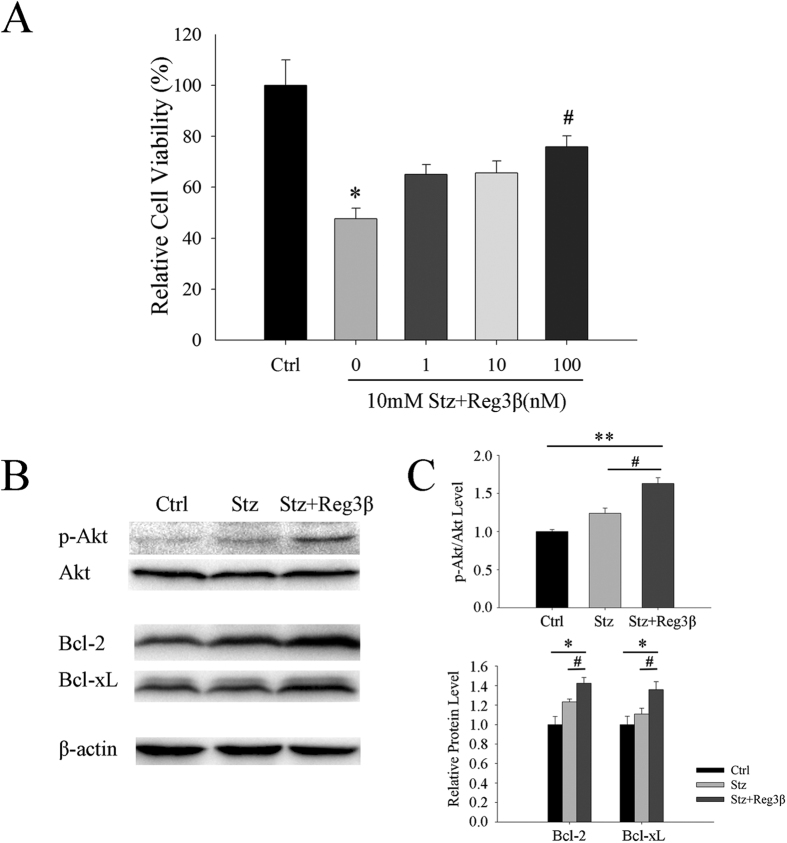
Recombinant Reg3β protein pretreatment protects primary mouse islets from Stz-induced cell death. (**A**) Effect on cell viability as measured by MTT. Cells were pretreated with 1–100 nM recombinant Reg3β protein for 12 h before Stz. N = 5; *p < 0.05 vs. Ctrl, ^#^*p* < 0.05 vs. Stz group using One Way ANOVA. (**B**) Changes in Akt phosphorylation and levels of Bcl-2 and Bcl-xL as determined by Western blot analysis. Images of phosphorylated and total Akt, and Bcl-2 and Bcl-xL are shown. (**C**) Densitometry results of the phosphorylated Akt and Bcl-2 and Bcl-xL levels. Some blots were edited for better representation and the full-length blots are included in the Supplementary Information file. For B and C, N = 3; *p < 0.05, **p < 0.01 vs. Ctrl, ^#^*p* < 0.05 vs. Stz group using One Way ANOVA.

To test the effect in normal islet cells, primary mouse islets were isolated and cultured for cell proliferation and survival assays. The resulting quantitative measurement of BrdU incorporation indicated no change in cell proliferation following pretreatment with recombinant Reg3β [Supplymentary Fig. 5]. On the other hand, the Stz-induced islet cell death was markedly inhibited by the pretreatment of 100 nM recombinant Reg3β, where cell viability increased from 48% to 76% [Fig. 7A]. At the same time we detected a significant 1.3 to 1.6 fold elevation in Akt phosphorylation and cellular Bcl-2 and Bcl-xL expression levels following treatment with recombinant Reg3β [Fig. 7B,C].

## Discussion

During the onset of diabetes, it has long been observed that a rapid and massive rearrangement of gene expression occurs in pancreas, including the Reg proteins^22^,^23^. A recent study using insulinoma cell lines in a mouse model provided biological evidence that β-cells undergoing cell death induce Reg protein expression in the surviving neighboring β-cells^9^. In our previous study, pancreatic β-cell-specific overexpressed Reg3β could resist Stz-induced diabetes^12^. We therefore produced a bioactive recombinant Reg3β protein to assess its therapeutic potential in the current study. Despite no curing effect on preexisting diabetes, pretreatment of recombinant Reg3β was effective in protecting against Stz-induced β-cell damage and diabetes. A possible mechanism for this protective effect was further explored and we believe that Reg3β stimulates Akt phosphorylation which in turn increases Bcl-2 and Bcl-xL levels, key factors in stabilizing the integrity of mitochondria and cell survival.

Stz-induced attacks on pancreatic islet β-cells leads to elevated production of nitric oxide (NO) and reactive oxygen intermediates (ROI), inducing widespread DNA strand breaks, PARP activation, and mitochondrial and ER stress^21^. DNA damage and some particular feedback mechanism can further interact with Reg gene transcription^24^. Nevertheless, the auto-induced Reg gene expression is unfortunately insufficient to achieve a protective outcome that will defend against islet β-cell destruction, even in Reg2-overexpressed transgenic mice^11^. Thus, we questioned whether there was any distinction between endogenous overexpression and exogenous administration of Reg protein affecting its bioactivities. The possibility that insufficient quantities of Reg protein, limiting its function, cannot be excluded. Given that Reg gene-related polypeptides, such as INGAP and its short fragment, have been proven effective in clinical trials^25^, we believe that treatment with recombinant Reg3β provides a promising and dosage controllable means to defend against the diseases of β-cell failure or eventual loss of β-cell mass.

In the present study, we found that pretreatment with recombinant Reg3β protected against Stz-induced diabetes in mice [Fig. 3], which is consistent with our previous study in transgenic mice^12^. The protective effect of recombinant Reg3β was mainly contributed by the preservation of serum and pancreatic insulin levels, and islet β-cell mass [Fig. 5 and Table 1]. The lack of statistical significance between the Stz and Stz + Reg3β groups could be owing to the existence of some mega islets and odd sectioning at different cut-off faces. Of note, the islet density or number of insulin-positive stained cell clusters, demonstrated an increase in both the Stz and Stz + Reg3β groups, yet no significant difference was observed between these groups [Table 1]. We speculate that this result may be due to the regeneration of insulin-producing cells after pancreatic islet injury^10^,^19^, and not Reg3β treatment. In our parallel study, recombinant Reg3α protein appeared to boost neogenesis and/or transdifferentiation of insulin-producing cells from pancreatic acinar- and ductal-cells after Stz induction, since numerous clusters of 1 ~ 5 insulin-positive cells were observed in the exocrine part of the pancreas (unpublished preliminary data). These data also suggest discrepant characteristics in the functions of the isoforms in the Reg gene family.

Although recombinant Reg3β was found to drive MIN6 cell proliferation via the Akt/ATF-2 pathway and upregulation of cellular cyclin D1 and CDK4 levels [Fig. 1], it failed to promote cell proliferation *in vivo* [Supplymentary Fig. 3]. It is known that MIN6 cells are transformed β-cells and proliferate at a much higher rate. At the same time, primary β-cells do not proliferate as much and respond poorly to stimuli. Although Akt activation has been known to be involved in cell proliferation, there are also situations that Akt phosphorylation was insufficient to drive cell proliferation^26^. In our study, Akt activation might *only* have provided a protective mechanism to the primary β-cells. To reach a similar level of stimulation as seen in MIN6 cells, we may need to use longer periods and higher doses of stimulation in order to sustain the proliferation signals, including Akt activation. This is likely to explain a possible reason for why recombinant Reg3β has no curing effect on preexisting diabetes [Fig. 4]. However, the current lack of efficiency in curing existing diabetes may also be caused by the limited dose, and the route and frequency of protein administration we have tested thus far. Even if the use of recombinant Reg3β protein is truly limited in its protective effects, it could still be used as a potential therapeutic agent as the development of diabetes mellitus in human differs from the current experimental models. In most cases, long diabetic onset duration provides strategic periods for medical interventions. In these periods, recombinant Reg3β can be used to prevent the secondary damage to islet β-cells caused by diabetic complications such as hyperglycemia; thus postponing or even helping to reverse the effects of diabetes.

The islet protective effect brought on by the pretreatment of recombinant Reg3β was confirmed in MIN6 cells, *in vivo* pancreatic sections, and primary mouse islets [Figs 2,5 and 7 and Table 1]. The three panels in Fig. 2A demonstrate an overall protection from Stz-induced cell death by 68% to only 15%, when combining early and late apoptosis, and necrosis together. Closer examination further suggests a Reg3β-induced *shift* from necrosis to late apoptosis then to early apoptosis (not by tracing individual cells but population-wise), which again supports a protective effect and is consistent with the data on Stz-treated primary β-cells. The Akt phosphorylation and increased Bcl-2 and Bcl-xL levels were also detected [Figs 2,6 and 7]. These results are in line with the evidence that Reg1 protein mediates an anti-apoptotic effect via activation of the Akt/Bad/Bcl pathway^27^, and that both recombinant Reg3α and Reg4 proteins protect against acinar-cell necrosis and apoptosis by the upregulating Bcl-2 and Bcl-xL levels^17^,^18^. To be noted, we have reported that Reg3β overexpression may prevent Stz-induced β-cell damage; due in part to a 20% reduction in cellular GLUT-2 expression^12^. GLUT-2 is the specific target of Stz action. While we cannot totally exclude that possibility, treatment with recombinant Reg3β did not cause the 10% elevation in basal glucose levels as seen with overexpression of Reg3β^12^. Whether it changes GLUT-2 levels is yet to be determined. Purified Reg3β (HIP/PAP/Reg-2) protein, however, has been reported to prevent TNF-α and actinomycin D-induced cell apoptosis, which is independent of GLUT-2^28^.

In this study, large amounts of Stz by single injection (150 mg/kg *in vivo* and 10 mM *in vitro*) triggered NO and ROI accumulation, and led to cell necrosis^29^. Immunofluorescent TUNEL staining showed that a few apoptotic cells were observed in the islets after Stz injection, suggesting that the massive insulitis-associated β-cell loss was predominantly caused by necrosis [Fig. 6]. In MIN6 cells, although late apoptotic cells cannot be distinguished from necrotic ones, our results clearly exhibited a decrease amongst the cells in late apoptotic/necrotic and necrotic phases after treatment with recombinant Reg3β [Fig. 2]. Whether the recombinant Reg3β protein protects against necrosis or apoptosis or both needs further experimentation. It is not difficult to show that necrosis leads to secondary damage, such as apoptosis, thereby suggesting that a drug enabling resistance to necrosis should be somehow capable of preventing apoptosis. Now that recombinant protein is available, we need to further elucidate its protective effects on other animal models such as NOD mice which resemble T1D more closely than Stz.

Reg genes encode extracellular growth factors featuring C-type lectin domains which activate multiple signaling pathways via their putative receptor exostosin-like glycosyltransferase-3 (EXTL-3)^30^. It further triggers Akt phosphorylation that is an upstream signal of cyclin D1, Bcl-2 and Bcl-xL expression^31^. Progression from G1 to S phase of the cell cycle requires activation of CDK4, which is regulated by the complex formation involving its catalytic partner cyclin D1. It has also been reported that disruption of the CDK4 gene results in the development of insulin-deficient diabetes due to a reduction in the number of islet β-cells^32^. Although the general knowledge of CDK4 regulation is achieved by its post-translational modification, and phosphorylation, we found that recombinant Reg3β stimulated its expression [Fig. 1] and this observation supports other reports involving Reg3α and Reg4^33^,^34^.

Of note, the use of recombinant Reg proteins might have uncertain side-effects. For example, Reg is involved in cancer and the human Reg4 gene has frequently been observed to be expressed in many gastric and intestinal malignancies, including gastric^35^, pancreatic^36^ and colorectal cancers^37^. Increased Reg4 gene expression in human colon cancer cell lines has also been reported to induce *in vitro* resistance to chemo- and radio-induced cell apoptosis^38^,^39^. Furthermore, a positive feedback loop involving Reg3α-JAK/STAT-3 has been confirmed to accelerate pancreatic cancer cell growth^40^. All these results suggest a possibility that the use of Reg proteins may disrupt cell growth and metabolism, and even worsen the course of oncogenesis. In this study, neither proliferation nor hypertrophy was found in islet α- or exocrine acinar-cells [Table 1, Supplymentary Figs 3 and 4], indicating that the use of recombinant Reg3β, at least under the conditions we tested thus far, was relatively safe. In the study of recombinant Reg2 protein, however, it served as an auto-antigen that worsened diabetes in BABL/c mice receiving multiple inductions with Freund’s adjuvant + Reg2 protein (preliminary unpublished data). The safety of using recombinant Reg2 needs further assessment.

## Conclusion

In the present study we present original evidence that recombinant Reg3β protein partially protected mice from Stz-induced β-cell damage and diabetes. Its underlying mechanism may include Akt phosphorylation and increased levels of Bcl-2 and Bcl-xL to resist β-cell death. On the other hand, recombinant Reg3β failed to increase primary β-cell proliferation, and alleviate preexisting diabetes. Furthermore, no marked change on the α- or acinar-cells was found by this treatment. In conclusion, we demonstrated that recombinant Reg3β protein protects against Stz-induced β-cell damage and diabetes, which provides a basis for studying its therapeutic potential.

## Materials and Methods

### Cell culture

Mouse insulinoma MIN6 cells were cultured in Dulbecco’s Minimum Essential Medium (DMEM, from WISENT) containing 25 mM glucose, 10% heat-inactivated fetal bovine serum (FBS, from WISENT) and 0.0005% 2-β-mercaptoethanol. Cells were seeded at a density of 10^6^ per well in 6-well plates, and then starved in the medium containing 1% FBS and 5.5 mM glucose for another 24 h. Then the vehicle, 10 nM and 100 nM of recombinant Reg3β protein were added and further incubated for 24 h. Cells were harvested for Western blotting and cell cycle assays using the Cell Cycle Kit according to the manufacturer’s instructions (Beyotime). In brief, the cells were washed with PBS and fixed by 70% ethanol. Propidium iodide (PI) was used to label DNA and red fluorescence was detected at the excitation wavelength of 488 nm for cell cycle analysis. The signals were captured by BD FACS Calibur and the data were performed using ModFit LT software.

### Western blotting

The cells were lysed in RIPA buffer (Sangon Biotech) containing 100 mM NaF, 10 nM sodium vanadate, 1 mM PMSF, and protease inhibitor cocktail (Pierce). The proteins were separated by 10% sodium dodecylsulfate (SDS)-polyacrylamide gel electrophoresis (PAGE), and electro-transferred to pure PVDF membranes (Millipore) at 200 mA for 1 h. The membranes were blocked at 37 °C for 1 h with 5% (w/v) non-fat milk powder in TBST (20 mM Tris, 0.9% NaCl, 0.1% Tween 20, pH 7.5), before being incubated with primary antibodies against β-actin (Sunbio Technology) diluted at 1:1000, and cyclin D1, CDK4, phosp-ATF-2, ATF-2, phosp-Akt, Akt, Bcl-2, and Bcl-xL (all from CST) diluted at 1:1000, at 4 °C overnight. Washed with TBST, the membranes were then incubated with HRP-conjugated secondary antibodies (Sunbio Technology), washed and incubated with a hypersensitive enhanced chemiluminescence reagent (Millipore). The luminescent signals were captured by Bio-rad ChemiDoc XRS+ System and densitometry quantification was performed using the AlphaEase software.

### Cell death assay

Cells were plated at a density of 10^6^ per well in 6-well plates and treated with 100 nM recombinant Reg3β or vehicle. Then, a final concentration of 10 mM Stz was added. Following a 24 h incubation period, cells were then harvested and washed with binding buffer and labeled with Annexin V-fluorescein isothiocyanate (FITC) and propidium iodide (PI), using the Annexin V-FITC Apoptosis Detection Kit (Vazyme) according to the manufacturer’s protocol. Normal cells and single-staining cells were used for gating and adjusting the compensation. Apoptotic and necrotic cells were captured by flow cytometry (FACS Calibur, BD), and the plots were analyzed by the FlowJo 7.6.1 software.

### Stz-induced diabetes in BALB/c mice

To induce pancreatic islet β-cell damage and diabetes mellitus, 12-week old, male BALB/c mice (Comparative Medicine Center of Yangzhou University), weighing 32 ± 2 g, were given a single intraperitoneal injection (i.p.) of Stz (Sigma) freshly prepared in citric acid-sodium citrate buffer at a dose of 150 mg/kg body weight after overnight fasting. Mice were maintained on a 12 h light-dark cycle at room temperature, fed on standard laboratory chow and water ad libitum. 2 d ***before*** the Stz injection, the recombinant Reg3β protein was administrated intravenously (i.v.) for 5 consecutive days at 100 μg/kg body weight. Random fed blood glucose and body weight were measured every 2 or 3 d using Roche ACCU-CHEK Performa glucometer and electronic balance. The areas under the curve (AUC) of individual mice were calculated by a trapezoidal method. Mice were sacrificed on the 15^th^ day for blood collection and pancreatic histological examination. Serum insulin levels were measured in random fed animals using the ELISA kits, according to the manufacturer’s protocol (Nanjing Jiancheng Bioengineering Institute). The relative pancreatic insulin content was measured using Image-Pro Plus 6.0 (Media Cybernetics), as reported before^41^.

Another set of animals (with the same age, sex and weight) were given Stz with a dose of 50 mg/kg per day i.p. for 5 consecutive days. Mice who steadily developed hyperglycemia (random fed blood glucose between 11~35 mmol/L on two consecutive days) were divided into two groups, in which one was provided with the recombinant Reg3β and the other with the vehicle. The recombinant Reg3β protein was administrated at 100 μg/kg i.v. twice a week all through the experiment. Random fed blood glucose and body weight was examined at each of the indicated time points. All experiments involving the use of animals and every handling procedure were performed in accordance with the guidelines and regulations set and approved by the Animal Care Committee of China Pharmaceutical University.

### Immunohistochemistry

The pancreatic tissue from each mouse was fixed in 4% paraformaldehyde and embedded in paraffin for sectioning. Pancreatic sections were dewaxed by xylene, rehydrated with ethanol, and permeabilized using 0.1% Triton X-100. Endogenous peroxidase was inactivated with 1.5% H_2_O_2_. The sections were blocked in 10% goat serum in PBS at room temperature for 1 h, followed by primary antibody incubation with anti-insulin and anti-glucagon (Abcam), and anti-Bcl-2 (CST), diluted at 1:200 in 1% goat serum at 4 °C overnight. Washed with PBS for 3 × 5 min, the sections were incubated with the HRP-conjugated secondary antibody at room temperature for 3 h, and reacted with diaminobenzidine substrate (DAB). Hematoxylin was stained for nuclei. Microscopic images were then captured using a Zeiss microscope and a 200 ×  and 400 ×  objective. The densitometry quantification of Bcl-2 was measured using the Image-Pro Plus 6.0 software.

### Immunofluorescence and TUNEL assay

The immunofluorescent double staining of insulin and glucagon, insulin and Ki67, and insulin and TUNEL were performed according to the standard procedures. In brief, the primary antibody incubation with anti-glucagon and anti-insulin were diluted at 1:200. The TUNEL signal was stained according to the manufacturer’s instructions (Bio-year Tech). Sections were then incubated with Cy3-conjugated secondary antibody (Sangon) and FITC-conjugated secondary antibody (Sunbio Technology), at 1:100 diluted in PBS at room temperature for 3 h in dark. The 4’, 6-diamidino-2-phenylindole (DAPI) was stained for nuclei. Microscopic images were then captured using a Zeiss microscope and a 400× objective. As the TUNEL signal was barely detected in the sections from the mice sacrificed on the 15^th^ day, another experiment with pancreatic tissues collected on the 2^nd^ day after Stz injection was performed for TUNEL assay.

### Pancreatic islet isolation

Pancreatic islets were isolated from 8–10-week old, male BALB/c mice using the collagenase digestion protocol, as reported previously^12^. The islet cells were then cultured in RPMI 1640 medium (WISENT) containing 25 mM glucose, and 20% heat-inactivated FBS (WISENT). For the cell proliferation assay, the islet cells were added every 24 h with fresh 1–100 nM recombinant Reg3β or vehicle and labeled by BrdU at 48 h. The rate of BrdU incorporation was quantified using the BrdU cell proliferation kit (Abcam). To study cell survival, the islet cells were pretreated with 1–100 nM recombinant Reg3β or vehicle for 12 h before being hit by 10 mM Stz for another 6 h. Cell viability was measured using the MTT assay (Sangon Biotech). Separate cells were also harvested for Western blotting to determine the phosphorylated and total Akt, as well as Bcl-2 and Bcl-xL levels.

### Statistical analysis

Data was expressed as mean ± standard error (SE) and analyzed by Student t-test or One Way ANOVA analysis using SigmaPlot version 11.0. A *p* value of <0.05 was considered as statistically significant.

## Additional Information

**How to cite this article**: Luo, C. *et al.* Recombinant Reg3β protein protects against streptozotocin-induced β-cell damage and diabetes. *Sci. Rep.* **6**, 35640; doi: 10.1038/srep35640 (2016).

## Supplementary Material

Supplementary Information

## Figures and Tables

**Table 1:** Statistical analysis of the pancreatic immunohistochemical sections.

	Control (5)	Stz (9)	Stz + Reg3β (9)	*p1*	*p2*
β-cell percentage (%)	0.45 ± 0.07	0.25 ± 0.03	0.37 ± 0.06	0.013	0.112
β-cell mass (mg)	1.04 ± 0.14	0.52 ± 0.07	0.79 ± 0.10	0.010	0.143
β-cell mass (mg/kg b. w.)	32.53 ± 4.49	20.16 ± 2.17	28.80 ± 3.33	0.053	0.133
α-cell percentage/islet (%)	12.54 ± 1.44	28.65 ± 1.92	21.44 ± 1.76	0.001	0.02
α-cell mass/pancreas (mg)	0.17 ± 0.03	0.23 ± 0.04	0.22 ± 0.04	0.327	0.862
average islet size(1000 × μm^2^)	9.22 ± 1.39	3.79 ± 0.67	6.73 ± 1.46	0.002	0.086
islet density (per cm^2^)	44.17 ± 2.16	59.59 ± 2.36	59.22 ± 4.52	0.001	0.943

Data was expressed as means ± SE and analyzed by one-way ANOVA using SigmaPlot version 11.0. The *p1* values were derived from the Stz group compared with Controls, while *p2* values indicated the differences between the Stz and Stz + Reg3β groups. A *p* value of <0.05 is considered statistically significant.
