# Supplementary material for: Recombinant Reg3β protein protects against streptozotocin-induced β-cell damage and diabetes
Source: Sci Rep. 2016 Oct 21;6:35640. doi: 10.1038/srep35640 (PMC5073304; doi:10.1038/srep35640)
Supplement: Supplementary Information [file srep35640-s1.doc]

Recombinant Reg3β protein protects against streptozotocin-induced β-cell damage and diabetes

Chen Luo ^a,b,1^, Lu-Ting Yu ^a,1^, Meng-Qi Yang ^a^, Xiang Li ^a^, Zhi-Yuan Zhang ^a^, Martin O Alfred ^a^, Jun-Li Liu ^c,*^, Min Wang ^a,b,*^

^a^ School of Life Science & Technology, China Pharmaceutical University, Nanjing, China; ^b^ State Key Laboratory of Nature Medicines China Pharmaceutical University, Nanjing, China; ^c^ Fraser Laboratories for Diabetes Research, Department of Medicine, McGill University Health Centre, Montreal, Canada

**^*^ Corresponding authors:** (1) Prof. Jun-Li Liu, Room E02.7220, RI-McGill University Health Centre, 1001 Décarie Blvd., Montreal, QC, Canada H4A 3J1. Phone: +1-514-934-1934 Ext 35059, Email: [jun-li.liu@mcgill.ca](mailto:jun-li.liu@mcgill.ca). (2) Prof. Min Wang, Room 1505, School of Life Science & Technology, China Pharmaceutical University, 24 Tong Jia Xiang, Nanjing, China 210009. Phone: +86-25-8327-1483, Email: [minwang@cpu.edu.cn](mailto:minwang@cpu.edu.cn).

**^1^ The authors** Chen Luo and Lu-Ting Yu contributed equally to this work.

**Supplementary Data**

**Suppl Fig. 1.**


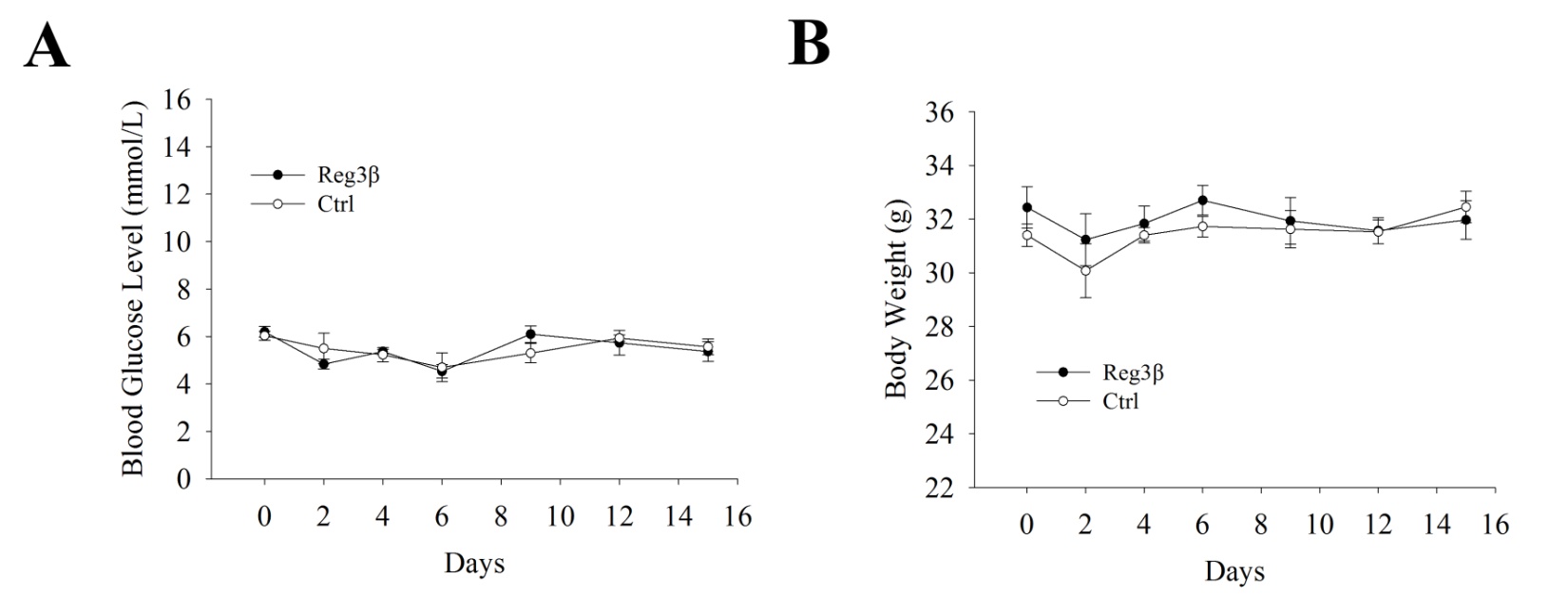


**Suppl Fig. 1. The blood glucose level and body weight were not affected by the treatment of recombinant Reg3β protein.** The protein was administrated intravenously for 5 consecutive days at 100 μg/kg body weight. N=4; no significant difference was found between the Control and Reg3β groups.

**Suppl Fig. 2.**


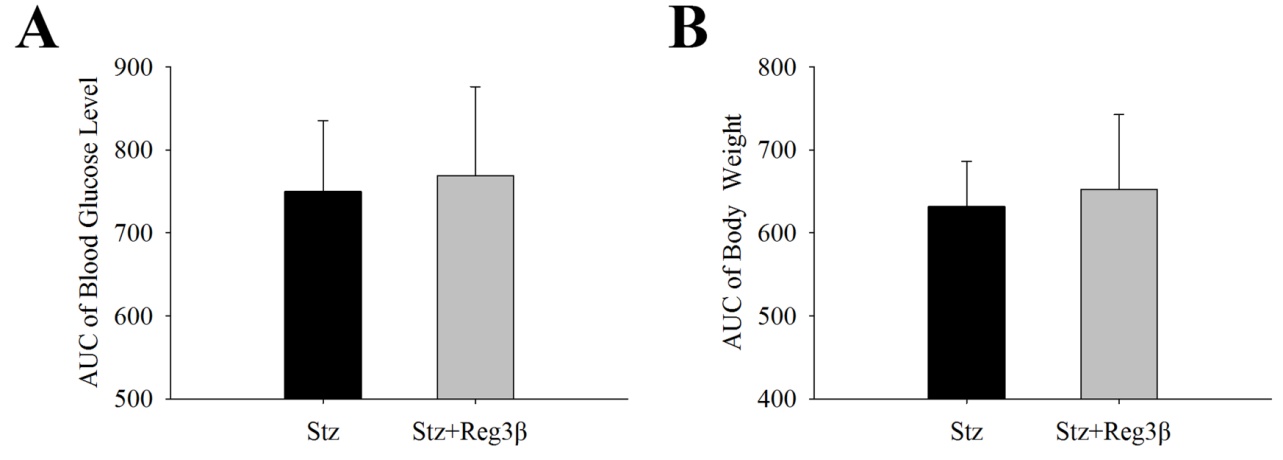


**Suppl Fig. 2. The AUC analysis of the blood glucose level and body weight in Figure 4.** N=9; no significant difference was found between the Stz and Stz+Reg3β groups.

**Suppl Fig. 3.**


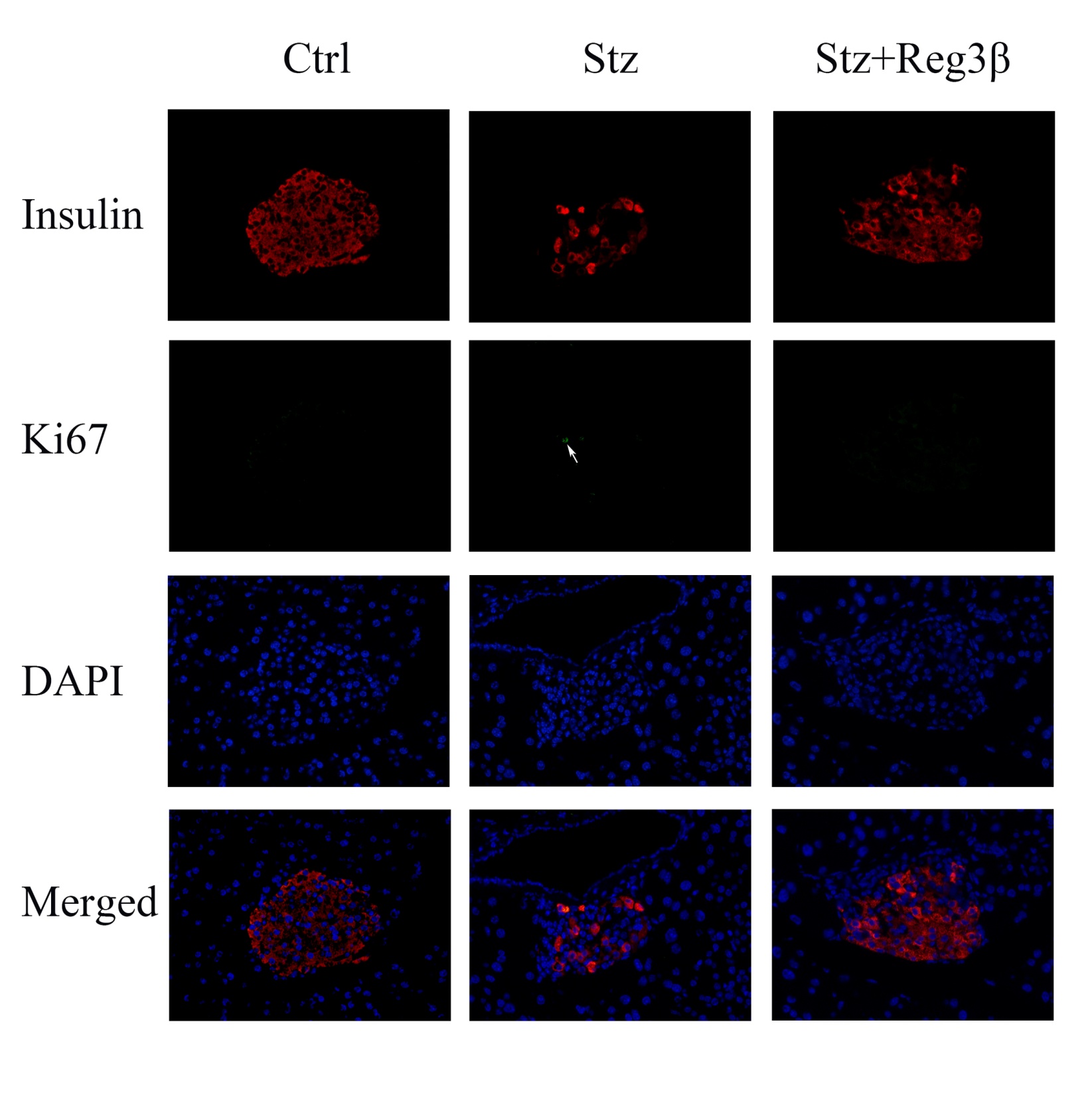


**Suppl Fig. 3. Immunofluoresecnce stained with insulin and Ki67 in the groups of Control, Stz and Stz+Reg3β.** Cell nuclei were labeled with DAPI. Arrow: Ki67 staining. A representative image was illustrated from each group. N=5-9; no significant difference was found between groups.

**Suppl Fig. 4.**


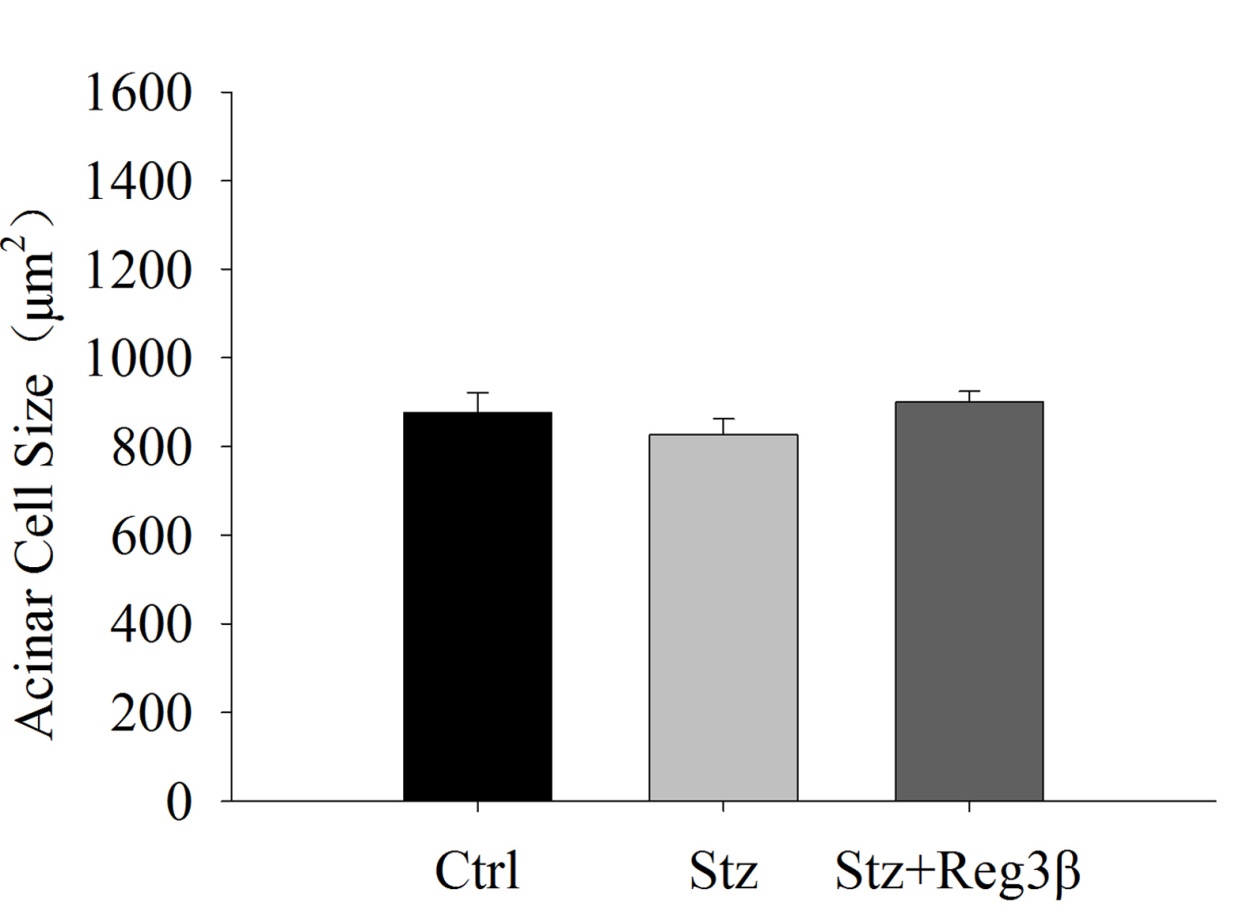


**Suppl Fig. 4. The average acinar-cell size in the groups of Control, Stz and Stz+Reg3β.** N=5-9; no significant difference was found between groups.

**Suppl Fig. 5.**


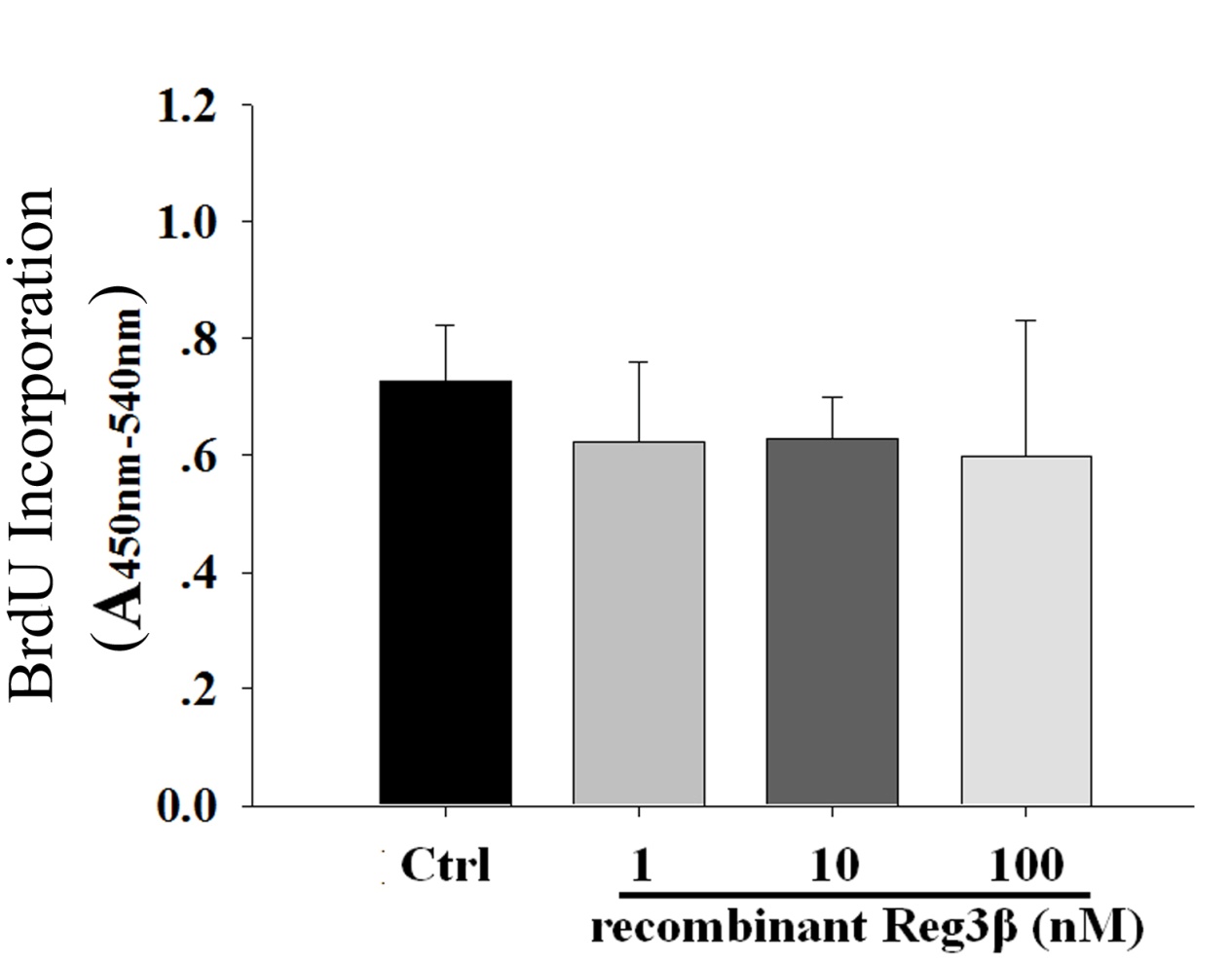


**Suppl Fig. 5. Effect of recombinant Reg3β pretreatment on the rate of cell proliferation measured by BrdU incorporation in primary islet cells.** N=5; no significant difference was found between the four groups.

**Supplementary Info** Some blots have been edited for better (clear cut) presentation. We have provided the original blots as much as we can.

**FIG.1. CyclinD1:**

**
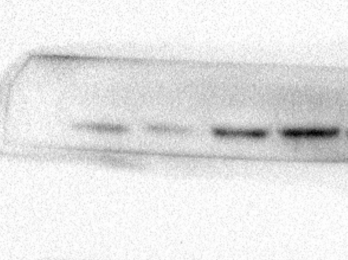
**

**FIG.1. CDK4:**

**
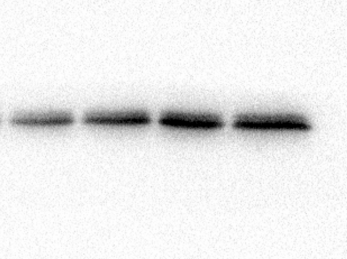
**

**FIG.1. β-actin：**

**
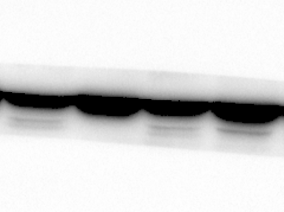
**

**FIG.1. p-ATF-2：**

**
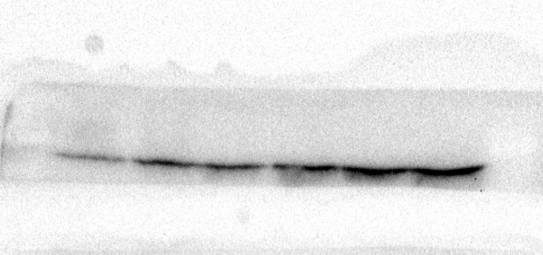
**

**FIG.1. ATF-2：**

**
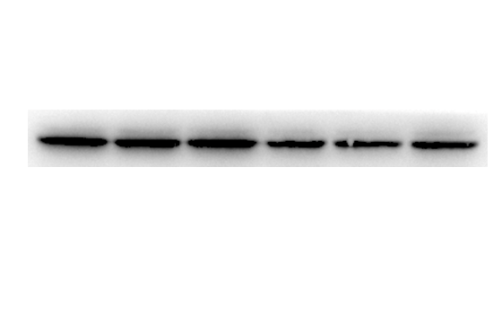
**

**FIG.1. β-actin：**

**
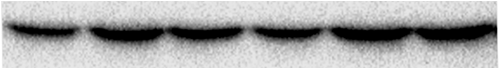
**

**FIG.2. p-Akt：**

**
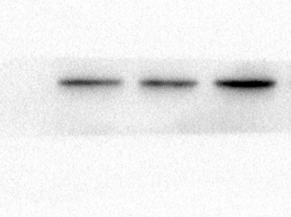
**

**FIG.2. Akt:**

**
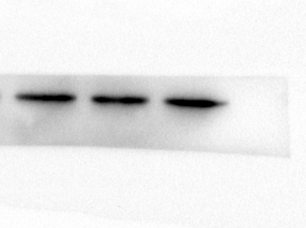
**

**FIG.2. Bcl-2:**

**
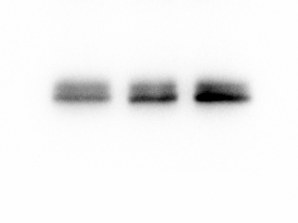
**

**FIG.2. Bcl-xL:**

**
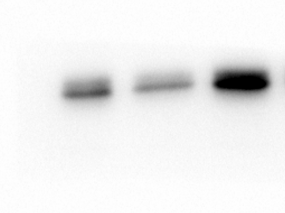
**

**FIG.2. β-actin：**

**
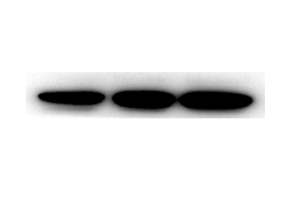
**

**FIG.7. p-Akt：**

**
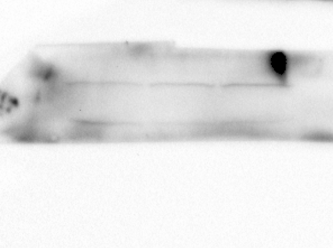
**

**FIG.7. Akt：**

**
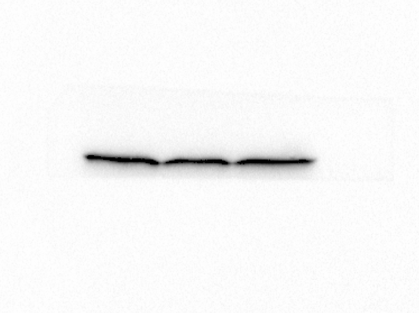
**

**FIG.7. Bcl-2：**

**
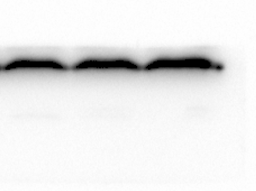
**

**FIG.7. Bcl-xL:**

**
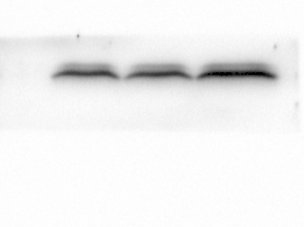
**
